# A Lithiophilic Artificial Li_3_P Interphase with High Li-Ion Conductivity via Solid-State Friction for Lithium Metal Anodes

**DOI:** 10.3390/ma18091930

**Published:** 2025-04-24

**Authors:** Haoling Liu, Wen Pan, Bo Xiao, Yunke Jin, Kun Li, An Wang, Huimiao Li, Zhibin Wu, Yuejiao Chen, Shaozhen Huang, Lin Mei, Libao Chen

**Affiliations:** State Key Laboratory of Powder Metallurgy, Central South University, Changsha 410083, China; lhl620@csu.edu.cn (H.L.); panwen@csu.edu.cn (W.P.); xiaobo2001@csu.edu.cn (B.X.); 223312102@csu.edu.cn (Y.J.); 8204211423@csu.edu.cn (K.L.); wangan@csu.edu.cn (A.W.); lihuimiao2019@csu.edu.cn (H.L.); zhibinwu@csu.edu.cn (Z.W.); cyj.strive@csu.edu.cn (Y.C.); lbchen@csu.edu.cn (L.C.)

**Keywords:** mechanochemical reaction, artificial interphase, solid-state friction, high Li-ion conductivity, lithium metal anode

## Abstract

Interfacial modification strategies for lithium metal anodes have emerged as a promising method to improve cycling stability, suppress lithium dendrite growth, and increase Coulombic efficiency. However, the reported chemical synthesis methods lead to side reactions and side products, which hinder their electrochemical performance. In this study, we propose a novel and facile red phosphorus-assisted solid-state friction method to in situ fabricate a uniform Li_3_P interphase directly on the surface of lithium metal. Interestingly, the as-formed artificial Li_3_P interphase with high ionic conductivity and lithium affinity features significantly enhanced interfacial stability and electrochemical kinetics. The symmetric cells based on Li@P with the Li_3_P interphase achieved a prolonged lifespan, over 1000 h, at 1 mA/cm^2^ with low polarization. When paired with a high-loading LiFePO_4_ cathode (10.5 mg/cm^2^), the Li@P||LiFePO_4_ full cell retained 88.9% of its capacity after stable cycling for 550 cycles at 2 C and further demonstrated the excellent performance and stability of the Li@P‖LiCoO_2_ full pouch cell. This study provides an efficient and scalable strategy for stabilizing lithium metal anodes, expanding new ideas for the development of next-generation high-energy-density batteries.

## 1. Introduction

Lithium metal anodes have been regarded as a promising candidate for next-generation high-energy-density batteries [1,2] due to their ultra-high theoretical capacity (3860 mAh g^−1^) and lowest electrochemical potential (−3.04 V vs. standard hydrogen electrodes) [3,4,5]. Compared to conventional graphite-based anodes, lithium metal can significantly enhance the energy density of batteries, making it an ideal choice for applications in electric vehicles and grid energy storage systems. However, the practical application of lithium metal anodes is severely hindered by several challenges, including uncontrolled dendritic lithium growth, low Coulombic efficiency, and heterogeneous charge transport at the interface etc. [6,7,8]. The inhomogeneous interface of lithium metal induces a non-uniform current distribution and space charge accumulation, resulting in the formation of uncontrolled lithium deposition [9]. Lithium dendrite growth, interface side reactions, and dead lithium formation collectively contribute to the low Coulombic efficiency of lithium metal anodes [10]. Therefore, addressing these interfacial issues is crucial for the successful application of lithium metal-based energy storage systems.

To address these interfacial issues, significant efforts have been made to enhance the stability and performance of lithium metal anodes, including surface modifications [11,12,13], electrolyte engineering [14,15,16], and structural designs of lithium foil [17,18,19]. Among the various approaches to improve the performance of lithium metal anodes, interfacial modification strategies have emerged as effective methods to enhance cycling stability and suppress lithium dendrite growth and are expected to achieve large-scale fabrication for practical applications. For instance, the incorporation of the Li_3_P/Cu coating layer on lithium metal with mixed ion/electron conductive features has been demonstrated to effectively enhance the uniform distribution of interfacial Li^+^ flux [20]. Theoretical calculations show that Li_3_P is a fast channel for lithium ions, which can reduce the diffusion barrier of lithium ions. The interconnected skeletons of Li_3_P/Cu with extensive active channels significantly enhance the electrochemical kinetics and promote the reversibility of Li plating/stripping processes. However, the electron conductive interface of lithium metal may result in Li^+^ accumulation on the surface of the coating layer, impeding the efficient accommodation of lithium. An alternative strategy involves constructing an inorganic/organic compound layer that is ionically conductive, ensuring that lithium deposition occurs preferentially on the surface of electrodes, thereby regulating the interfacial Li^+^ distribution. The mechanically enhanced hybrid inorganic/organic coating for lithium metal anodes has been reported [21]. The electrically insulating polymer on the coating surface can confine the electro-deposition of Li and can tolerate volume changes, while inner inorganic lithiophilic sites can effectively facilitate and regulate uniform Li nucleation and deposition. Nevertheless, these structures may suffer from large interfacial impedance and sluggish electrochemical kinetics. Therefore, achieving a homogeneous distribution of ions and electrons is essential for stabilizing lithium metal anodes while enhancing electrochemical performance.

Li_3_P has emerged as a highly promising interfacial modification layer for lithium metal anodes due to its high ionic conductivity [22] and lithiophilicity [23]. Li_3_P enables fast lithium-ion transport while simultaneously acting as a protective barrier to suppress undesirable side reactions at the lithium metal–electrolyte interface. Tu et al. [24] utilized an evaporation–condensation technique to create an ultrathin P nanolayer composite (P-S-graphite) on the graphite surface, leveraging the bridging capability of S molecules. During the battery activation process, P undergoes an irreversible electrochemical reaction to form Li_3_P, resulting in the creation of an Li_3_P-based solid electrolyte interphase (SEI) on the anode surface. The robust interaction between Li^+^ and Li_3_P, along with its high ionic conductivity, promotes an efficient Li^+^ desolvation process and swift Li^+^ migration within the SEI film, thus enabling the battery to achieve fast-charging capabilities. Li_3_P serves as a chemically and mechanically robust artificial interface, significantly improving the interfacial stability of lithium metal anodes [25]. Despite the progress made in the Li_3_P interface of lithium metal anodes, many reported chemical synthesis methods suffer from undesired side reactions and side products, leading to phase impurities that hinder electrochemical performance. The development of novel interfacial engineering techniques, particularly in situ reactions, deserves significant attention. Recently, researchers have introduced an innovative solvent-free brush coating technology, which utilizes the reactivity between the powder and the lithium metal to achieve in situ lithiation of the powder directly on the surface of the Li foil, thereby generating a functional layer that regulates the interface stability of the negative electrode [26]. This is a promising surface modification strategy.

In this study, we propose a facile red phosphorus-assisted solid-state friction method to in situ fabricate a uniform Li_3_P interphase directly on the surface of lithium metal. The strong interaction between phosphorus and lithium leads to the formation of a lithiophilic Li_3_P interface, which promotes uniform lithium nucleation and dense lithium deposition. This lithiophilic nature ensures a more compact and dendrite-free lithium morphology, minimizing the risk of short circuits and improving the safety of the battery. The Li@P anode demonstrates excellent electrochemical performance, with an incredibly long lifespan exceeding 1000 h (1 mA/cm^2^; 1 mA h/cm^2^). Notably, the Li@P||LiFePO_4_ full cell retains a remarkable capacity retention of 89% after 550 cycles at 2 C. Additionally, the 0.2 Ah Li@P‖LiCoO_2_ pouch cell remains at 0.17 Ah capacity after 150 cycles, maintaining 89.5% capacity. The Li_3_P interphase obtained through the red phosphorus-assisted solid-state friction method improves the lithium deposition behavior and extends the cycling lifespan of lithium metal batteries.

## 2. Materials and Methods

### 2.1. Materials

Red phosphorus (P, ≥99.999% metals basis) powder was purchased from Shanghai Aladdin Biochemical Technology Co., Ltd. (Shanghai, China). Commercail Li foil, with a thickness of 100 μm and 50 μm, was purchased from Tianjin Zhongneng Lithium Industry Co., Ltd. (Tianjin, China). Commercial LiFePO_4_ (LFP) and commercial LiCoO_2_ (LCO) cathode electrodes were provided by Guangdong Canrd New Energy Technology Co., Ltd. (Guangzhou, China).

### 2.2. Fabrication of Li@P

**Figure 1:**
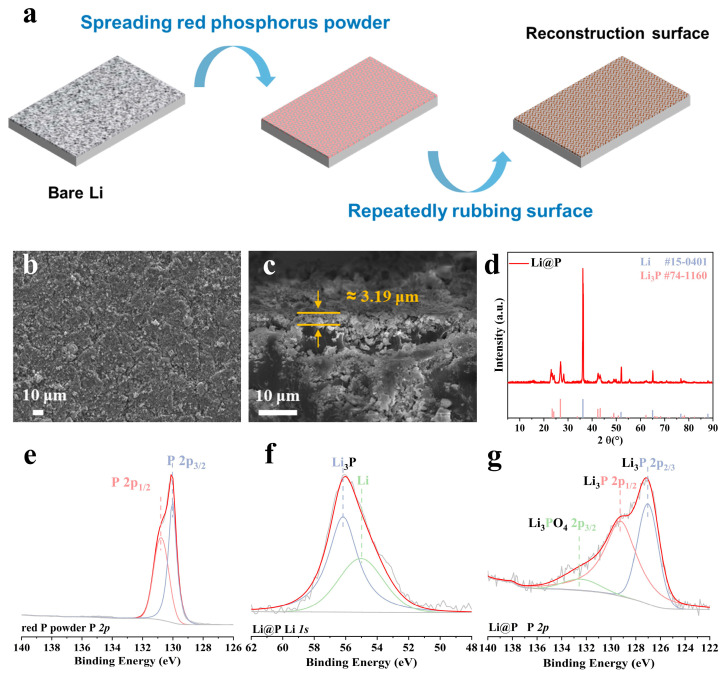
(**a**) Diagram depicting the manufacturing procedure of Li@P; (**b**) front SEM image of Li@P; (**c**) cross-sectional SEM images of Li@P; (**d**) X-ray diffraction (XRD) pattern of the as-prepared Li@P; (**e**) XPS spectra of red phosphorus powder: P 2p; XPS spectra of Li@P: (**f**) Li 1s and (**g**) P 2p.

Figure 1a schematically illustrates the mechanochemical surface reconstruction of Li foil. The fabrication of Li@P was carried out in an argon-filled glove box, where approximately 15 mg of red phosphorus powder was evenly applied to the surface of a Li foil (8 cm × 5 cm). Using nitrile gloves, the powder was manually rubbed onto the Li foil until the surface color changed to dark grey (Figure S3), confirming the lithiation of red phosphorus via a mechanochemical reaction. Excess powder was subsequently removed, resulting in a homogeneous interface layer with a loading density of approximately 0.045 mg/cm^2^.

### 2.3. Material Characterization

A normal scanning electron microscope (SEM, JEOL JSM-7610FPlus equipped with an Oxford ULTIM MAX 40 energy spectrometer, Tokyo, Japan) and a vacuum transfer accessory, provided by KW-ST Lab (www.kewei-scitech.com), were used to visualize the micromorphology. The chemical composition of the red phosphorus powder and Li@P anodes were analyzed by X-ray photoelectron spectroscopy (XPS, Thermo Scientific K-Alpha+, Waltham, MA, USA). When testing the Li metal samples, the samples were sealed in an in situ test mold to avoid contact with the air. The contact angles of liquid electrolytes (10 μL droplets) on the surface of the Li electrodes were measured at 25 °C on a Contact Angle System OCA 20 (Dataphysics, Filderstadt, Germany) in a drying room.

### 2.4. Electrochemical Measurements

To investigate the electrochemical behavior of Li@P electrodes, Li||Li symmetrical cells, Li||LFP button full cells, and 0.2 Ah Li||LCO pouch cells were assembled. The discharge or charge measurements were conducted on the Neware battery test system or the Land battery test system. Li||Li symmetrical cells and Li||LFP button full cells were assembled using a CR2016 button cell, using Celgard 2400 as the separator (Charlotte, NC, USA). A 0.10 mm Li foil and Li@P with a diameter of 14.0 mm were employed as the anode materials. The electrolyte used for the symmetrical batteries was 1.0 M of LiTFSI in a mixture of 1,3-dioxalane and dimethyl ether (1:1 by volume) with a 2 wt% LiNO_3_ additive, while the electrolyte used for the LFP full batteries and Li||LCO pouch cells was 1.0 M of LiPF_6_ in EC:EMC (3:7 *v*/*v*) with 10% FEC. The electrolyte injection volume for the coin cells was uniformly set to 45 μL. A high-load LiFePO_4_ cathode electrode (approximately 10.5 mg/cm^2^) was obtained from Dongguan Juda Electronics Co., Ltd. (Ningbo, China). The full cells were cycled between 2.5 and 4.2 V at 0.5 C for the first three cycles and were further cycled at different rates for the galvanostatic cycle test, where 1 C = 170 mA/g. The 0.2 Ah Li||LCO pouch cells were obtained by a lamination process, which was stacked by one piece of a commercial LCO cathode electrode (areal LCO loading of 35.2 mg/cm^2^ (double-sided)) with a size of 5.5 cm × 7.9 cm, two pieces of a 100 μm-thin lithium metal anode electrode with a size of 5 cm × 8 cm, and a piece of a 15 μm-thin PE separator. The Li||LCO pouch cells were discharged to 3.0 V at 0.5 C and charged to 4.3 V at 0.3 C for the cycle test (1C = 160 mA/g). Electrochemical impedance spectroscopy (EIS) was performed in the frequency range of 100 kHz to 0.1 Hz, with an alternating voltage amplitude of 5 mV. The Tafel curves of the Li||Li symmetrical cells were measured at a scan rate of 1 mV/s from −0.2 to 0.2 V. The exchange current density was calculated based on the Tafel equation. A chronoamperometry (CA) test of the Li||Li symmetric cells was conducted at a constant voltage of 150 mV for 1800 s. The CV curve and Tafel plots were generated through linear sweep voltammetry within the voltage range of −0.15 V to 0.15 V at a scan rate of 0.1 mV/s. The EIS, Tafel curves, CA, and CV were recorded at an electrochemical workstation (Ivium, Eindhoven, The Netherlands).

## 3. Results and Discussion

### 3.1. Compositional Analysis of Li_3_P Interphase via Solid-State Friction

Figure 1a illustrates the schematic process of coating red phosphorus powder onto the surface of a lithium strip and repeatedly mechanically rubbing the surface of the lithium strip to construct a protective layer on the lithium strip surface. The X-ray diffraction (XRD) pattern of the pristine red phosphorus powder is presented in Figure S1. A microscopic image of the as-received red phosphorus powder after milling, obtained via scanning electron microscopy (SEM), is shown in Figure S2. The optical photographs of the composite lithium strip after surface treatment are presented in Figure S3. The Energy Dispersive Spectroscopy (EDS) spectrum of the modified electrode is depicted in Figure S4. It is evident that the protective layer on the surface of the lithium strip is relatively uniformly attached and exhibits a certain degree of roughness (Figure 1b), with the entire protective layer being approximately 3.19 μm thick (Figure 1c). The enthalpy change is −312.366 kJ/mol for Equation (1):

3 Li(s) + P (s) → Li_3_P (s) (1)

This illustrates that the reaction has a strong driving force and is more likely to occur at a normal temperature and pressure. To determine the phase composition of the fabricated Li@P anode, X-ray diffraction (XRD) analysis was performed on the protective layer scraped from the electrode surface, as illustrated in Figure 1d. The six diffraction peaks at 23.5°, 24.1°, 26.8°, 42.4°, 43.4°, and 49.3° match well with the Li_3_P phase (PDF#74-1160). And the peaks of 36.2°, 51.9°, 64.9°, 76.7°, and 87.9° correspond well to the standard Li phase (PDF #15-0401). As a result, the prepared surface layer of Li@P is proved to be composed of the Li_3_P compound. The Fourier-transform infrared spectroscopy (FT-IR) results of the red phosphorus powder and Li@P anode are presented in Figure S5. The change of functional groups also proves the occurrence of Li and P reactions. The X-ray photoelectron spectroscopy (XPS) was used to analyze the original red phosphorus powder and the prepared Li@P electrode. Figure 1e shows that the P 2p_1/2_ and P 2p_3/2_ peaks in the original red phosphorus powder correspond to 131.1 eV and 130.3 eV [27,28], respectively. Analysis of the Li 1s spectrum (Figure 1f) reveals that the Li_3_P peak and the Li peak correspond to 56.1eV and 55.1 eV [29,30], respectively. Additionally, the P 2p spectrum in Figure 1g shows that Li_3_PO_4_ 2p_1/2_, Li_3_P 2p_1/2_, and Li_3_P 2p_3/2_ correspond to 132.4 eV, 129.0 eV, and 127.0 eV [23,31,32], respectively, demonstrating that a phosphide interface composed of the compounds sparing Li_3_PO_4_ and Li_3_P was formed on the surface of the Li@P electrode. The results demonstrate that the interface phosphating progress was achieved after modification.

### 3.2. Electrochemical Properties of Li_3_P Interphase via Solid-State Friction

**Figure 2:**
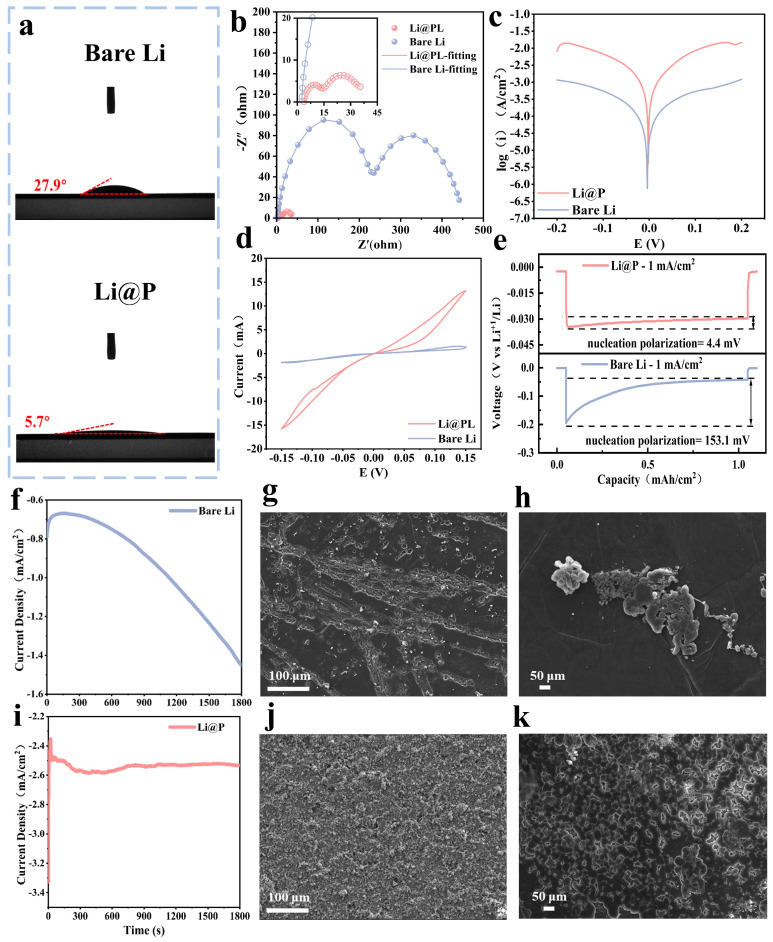
(**a**) The contact angles of bare Li and Li@P with ether-based electrolyte; (**b**) electrochemical impedance spectroscopy (EIS) plots before batteries’ cycling at room temperature, built using bare Li and Li@P; (**c**) Tafel curves of symmetrical cells built using bare Li and Li@P; (**d**) cyclic voltammetry (CV) curves of symmetrical cells built using bare Li and Li@P; (**e**) nucleation overpotential of bare Li and Li@P symmetrical cells at 1 mA/cm^2^ in the first cycle; chronoamperometry (CA) at 150 mV of overpotential for (**f**) bare Li and (**i**) Li@P; the CA stripping and deposition morphology of (**g**,**h**) bare Li electrode and (**j**,**k**) Li@P electrode.

Contact angle tests were conducted on both bare lithium anodes and Li@P anodes using the ether-based electrolyte (LS-009), with the results depicted in Figure 2a. The wetting angle measurements of the ester-based electrolyte (LB-515) on two electrodes are shown in Figure S6. The contact angle of the electrolyte on the Li@P anode was measured at 6.0°, significantly lower than that on the bare lithium anode at 35.5°. Compared to bare lithium, the Li@P anode demonstrates reduced contact angles with all tested electrolytes, suggesting enhanced electrolyte–electrode interfacial compatibility. In comparison to bare Li, the electrode’s interface, subjected to rubbing treatment, exhibits enhanced roughness, thereby fostering improved wettability at the reconstructed interface with the electrolyte. This roughness translates into an expanded electrochemical reaction surface area, which advantageously reduces the actual current density and mitigates dendrite growth issues stemming from high current densities [33,34].

Figure 2b presents the electrochemical impedance spectroscopy (EIS) analysis results of the symmetric cells with different anodes. Generally speaking, the Nyquist spectra of lithium metal batteries show two different characteristics: a semi-circular arc at a high frequency and an intermediate frequency and a diagonal line at a low frequency. These characteristics reflect the charge transfer resistance (R_ct_), the impedance related to the solid electrolyte interface film (SEI) (R_SEI_), and the Warburg impedance (W), respectively. An equivalent circuit diagram is shown in Figure S7 [35]. Table S1 compares the fitted impedance results of this work with the reported data from other surface-modified electrodes. Notably, the Li@P symmetric cell demonstrates significantly lower interfacial resistances, with a solid electrolyte interphase (SEI) resistance (R_SEI_) of merely 8.3 Ω and a charge transfer resistance (R_ct_) of 24.7 Ω. These values represent an order-of-magnitude reduction compared to the bare lithium symmetric cell (R_SEI_ = 209.7 Ω; R_ct_ = 223.7 Ω). In addition, compared with the impedance data of other interface-modified anodes, the results of this work also have advantages [36]. This is attributed to the high Li^+^ conductivity of Li_3_P, which supports the rapid diffusion of Li^+^ at the interface. To investigate the charge transfer kinetics at the anode, symmetrical cells were assembled for a Tafel test. The Tafel tests results, illustrated in Figure 2c, reveal that the exchange current density of the Li@P anode is 1.795 mA/cm^2^, which is higher than that of the bare lithium anode at 0.165 mA/cm^2^, indicating that the constructed protective layer, comprising Li_3_P, enhanced the electrochemical kinetics in the Li@P anode. The improvement in ion transport and charge transfer kinetics led to a reduction in the lithium-ion concentration at the electrode surface [37], which decreased the local current density and enhanced ion and charge mobility. This optimization facilitated uniform lithium deposition, effectively suppressing dendrite formation. Furthermore, the ionic conductivity of the Li@P anode was investigated through electrochemical characterization by assembling the symmetrical cells with Li@P and bare lithium as a control sample. As shown in Figure 2d, the cyclic voltammetry (CV) curves reveal that the Li@P cell demonstrates a significantly higher current peak within the same voltage window compared to the bare lithium cell. This result suggests that the Li@P material possesses enhanced ionic conductivity and superior reaction kinetics during electrochemical processes [38]. The rubbing treatment process removes the passivation layer on the commercial lithium foil while forming a lithiophilic reconstruction layer. Therefore, the nucleation overpotential of the Li@P anode during the initial stripping process is 4.4 mV, which is markedly lower than that of the bare lithium anode at 151.3 mV (Figure 2e), indicating a reduced nucleation barrier and better lithiophilicity of the Li@P anode interphase.

To investigate the impact of Li foil surface reconstruction on Li deposition/stripping behavior, chronoamperometry (CA) tests were performed on symmetric cells (Figure 2f–k). Under a constant voltage, electrochemical reactions initiate at the active sites on the Li electrode interface, generating a current density–time curve. Based on the Butler–Volmer equation [39], this curve reflects morphological changes at the electrochemical interface during Li deposition/stripping. Specifically, interfacial instability (e.g., non-uniform Li stripping and dendrite growth) leads to an expanding reaction area, causing the current density to rise continuously. In contrast, a stable interface results in a steady or decreasing current density. The CA test results in Figure 2f reveal that the bare Li anode displays a near-linear current increase, indicating persistent interfacial instability throughout the test. The CA curve of the Li@P anode recovers to a stable level within about 300 s, indicating the rapid stabilization of surface roughness. The distinct phenomena exhibited by the bare Li anode and Li@P anode originate from the heterogeneous distribution of active sites across their electrochemical interfaces. A high density of lithiophilic Li_3_P was uniformly distributed across the Li@P anode interface, acting as active sites for electrochemical lithium deposition. This confirmed a more uniform and dense Li deposition. The SEM images of the bare Li and Li@P surface morphologies after CA testing are shown in Figure 2g,h,j,k, which provide further evidence supporting the above conclusions. The bare lithium anode develops many dendrites and exhibits significantly non-uniform lithium-ion deposition during cycling. On the contrary, the Li@P anode shows uniform and dense lithium-ion deposition. In summary, the artificial interface layer on modified lithium foil enabled uniform and dendrite-free lithium deposition due to two key factors: (1) the high ionic conductivity of Li_3_P facilitated rapid lithium-ion transport and diffusion at the interface, and (2) the strong lithiophilic nature of Li_3_P provided abundant active sites for lithium-ion deposition, promoting uniform lithium plating and enhancing the charge transfer kinetics [40,41].

**Figure 3:**
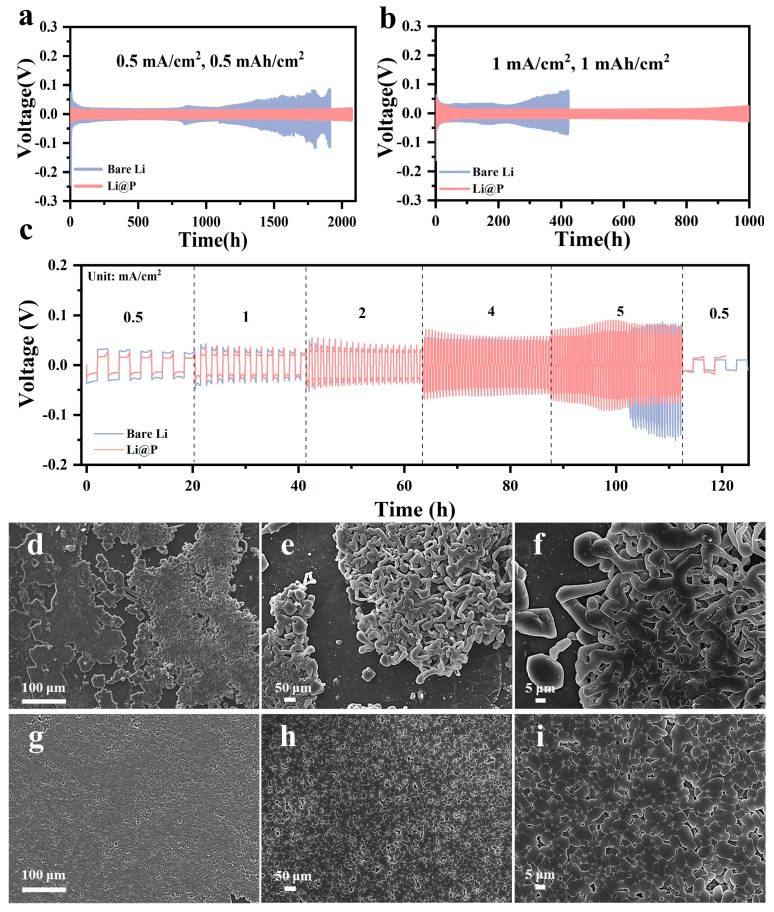
Voltage–time curve of the Li||Li symmetric cells in a 1 M LiPF6 in DME/DOL (*v*/*v* = 1:1) electrolyte with 2.0 wt% LiNO_3_ at a current density of (**a**) 0.5 mA/cm^2^ and a capacity of 0.5 mA h/cm^2^ (**b**) 1.0 mA/cm^2^ and a capacity of 1.0 mA h/cm^2^; (**c**) voltage profiles of symmetric cells at different current densities, from 0.5 to 5 mA/cm^2^, with a fixed capacity of 1 mA h/cm^2^; SEM images of (**d**–**f**) bare Li and (**g**–**i**) Li@P after the 50th cycle in Li||Li symmetrical cells at 1 mA/cm^2^ and 1 mA h/cm^2^.

Owing to the significant enhancement in the electrochemical stability of the Li@P anode by the phosphide protective layer, long-term characterization of symmetric cells composed of different anodes was conducted to evaluate their cycling performance and durability. The results, as depicted in Figure 3a,b, demonstrate that the Li@P||Li@P symmetric cells achieved exceptional stability, maintaining smooth and consistent voltage profiles for over 2100 h at a current density of 0.5 mA/cm^2^. This remarkable performance, equivalent to 1050 long-term cycles, underscores the effectiveness of the Li_3_P interphase in stabilizing the lithium metal anode. In contrast, symmetric cells using bare lithium electrodes exhibited severe voltage fluctuations and a much shorter lifespan, typically failing within a few hundred hours due to dendrite formation and electrolyte degradation. Under the same conditions, the polarization voltage of the bare lithium significantly increased after 550 cycles, leading to failure. The superior cycling stability of the Li@P||Li@P symmetric cell can be attributed to the uniform and lithiophilic Li_3_P interphase, which not only promotes homogeneous lithium-ion deposition but also mitigates the formation of dendrites and dead lithium. The protective layer acts as a robust barrier, preventing direct contact between the lithium metal and the electrolyte, thereby reducing side reactions. Additionally, the Li_3_P interphase enhances the mechanical strength of the electrode, enabling it to withstand the repeated volume changes that occur during lithium plating and stripping. This results in a more stable electrode–electrolyte interface and a prolonged cycle life. At a current density of 1 mA/cm^2^, Li@P also maintained stable cycling for 1000 h and 500 cycles, exhibiting a substantial improvement in cycling duration and stability compared to bare lithium.

The rate capability of the symmetrical cells was evaluated at various current densities ranging from 0.5 to 5 mA/cm^2^ with a fixed areal capacity of 1 mA h/cm^2^. As illustrated in Figure 3c, the Li@P electrode exhibits significantly improved performance compared to bare lithium, particularly at lower current densities (0.5–2 mA/cm^2^), as evidenced by its reduced polarization voltage. This lower polarization directly reflects enhanced electrochemical kinetics and superior cycling stability, both of which are essential for developing high-performance lithium metal batteries. The improved performance can be attributed to the uniform Li_3_P phase formed on the Li@P electrode, which facilitates more efficient lithium-ion transport and reduces interfacial resistance. In contrast, the bare Li electrode exhibited higher polarization, suggesting greater kinetic limitations and potential instability during cycling. Notably, it shows no distinct performance advantage at a high current density (4 and 5 mA/cm^2^). During the charge–discharge cycle of the cell, the Li@P-modified layer with electronic conductivity serves as a deposition layer and facilitates the transport of lithium ions. When the current density is substantial, the limited electrochemical reaction active sites are covered by freshly deposited lithium, and the reconstructed layer is unable to offer effective assistance in the subsequent plating process. Microstructural characterization of the two electrodes after cycling provided further insights into their performance differences. As shown in Figure 3d–f, the bare lithium electrode developed a large number of randomly distributed dendrites, which are known to penetrate the separator and cause short circuits, leading to safety hazards and capacity degradation. These dendritic structures are a direct consequence of uneven lithium-ion deposition and the absence of a protective layer to regulate ion flux. In stark contrast, the Li@P electrode exhibited a more uniform and dense lithium-ion deposition morphology after cycling, as shown in Figure 3g–i. This homogeneous deposition is a result of the lithiophilic Li_3_P interphase, which not only promotes uniform lithium nucleation but also effectively suppresses dendrite formation by redistributing the ion flux across the electrode surface. Compared with the spherical lithium deposits in Figure 3i, lithium dendrites (Figure 3f) have a larger surface area and consume more electrolytes. In addition, the fragile dendrites shedding from the lithium substrate will form a ‘dead lithium’ that does not participate in the charging and discharging process, reducing the Coulomb efficiency of the battery. The phosphide protective layer plays a crucial role in enhancing the long-term cycling stability of the battery. By inhibiting dendrite growth and ensuring uniform lithium-ion deposition, the Li@P electrode maintains structural integrity and minimizes capacity loss over extended cycles. This improvement in deposition behavior is particularly important for practical applications, as it addresses one of the key challenges associated with lithium metal anodes. The findings highlight the potential of the red phosphorus-assisted solid-state friction method to create advanced electrode materials with superior performance and safety, paving the way for the development of next-generation lithium metal batteries.

### 3.3. Full Cell Tests for Practical Applications

**Figure 4:**
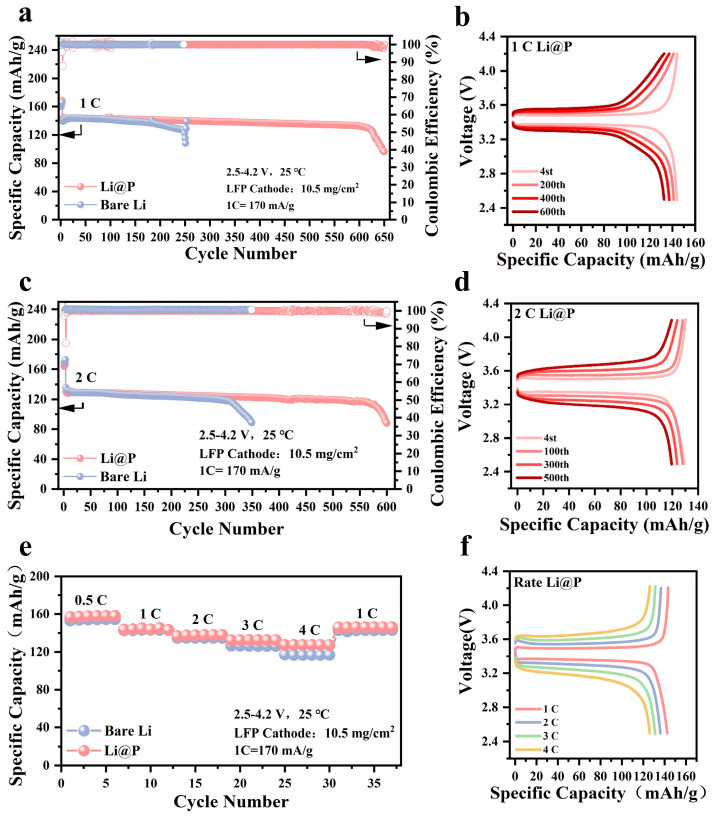
Full batteries’ performances based on Li@P anodes. (**a**) Cycling performance of the Li||LFP and Li@P||LFP full cells using 1.0 M of LiPF6 in EC:EMC:FEC= 3:7:1 Vol% electrolytes at a 1 C rate; (**b**) the charge–discharge profiles of Li@P||LFP at a 1 C rate after cycling; (**c**) cycling performance of the Li||LFP and Li@P||LFP full cells at a 2 C rate; (**d**) the charge–discharge profiles of Li@P||LFP at a 2 C rate after cycling; (**e**) cycling performance of the Li||LFP and Li@P||LFP full cells at different rates; (**f**) the charge–discharge profiles of Li@P||LFP at different rates after cycling.

**Figure 5:**
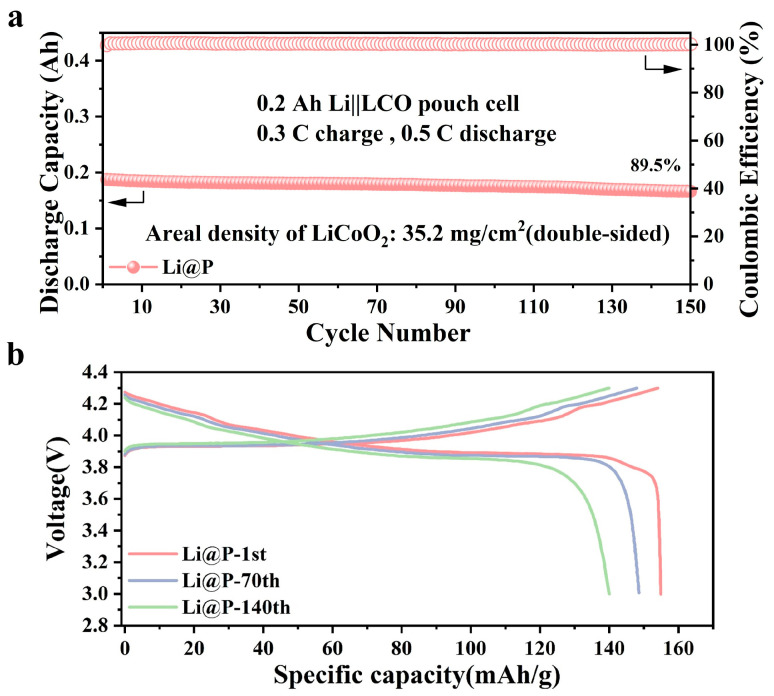
Electrochemical performance of practical 0.2 Ah Li@P‖LiCoO_2_ pouch cells with Li@P anode; (**a**) cycling performance under 0.3 C charge and 0.5 C discharge; (**b**) the voltage profiles of Li@P‖LiCoO_2_.

To validate the practicality of the Li@P anode, full cells comprising Li||LFP and Li@P||LFP were assembled, utilizing LiFePO_4_ as the cathode material, to further evaluate their electrochemical performance. All full cells were initially subjected to three activation cycles at a rate of 0.1 C. As illustrated in Figure 4a,b, the Li@P||LFP full cell, which exhibited an initial discharge specific capacity of 145.4 mA h/g, maintained a capacity retention rate of 91% after 600 cycles at a current density of 1 C. In contrast, the specific capacity of the Li||LFP full cell significantly declined to failure after 250 cycles. The capacity–voltage profiles of the Li||LFP full cell at rates of 1 C and 2 C are depicted in Figures S8 and S9. When the current density was increased to 2 C, the Li@P||LFP full cell demonstrated a slower capacity decay rate (from 132.4 mA h/g to 117.6 mA h/g) during cycling, retaining 88.9% of its capacity after 550 cycles. This represents a substantial improvement in cycle life and stability compared to the Li||LFP full cell, which retained only 65.3% of its capacity after 350 cycles (Figure 4c,d). The Li@P anode also exhibited superior performance in rate capability tests, outperforming the bare lithium anode across discharge rates ranging from 0.5 C to 4 C (Figure 4e,f). The discharge rates, ranging from 0.5 C to 4 C, of the bare lithium anode are depicted in Figure S10. The results further substantiate the claim that the Li@P anode possesses excellent cycling stability and high-rate tolerance, providing valuable insights into achieving high reversible capacity and long-term stable cycling in lithium metal batteries. The 0.2 Ah pouch cell, assembled with the 50 μm Li@P anode and a commercial LiCoO_2_ cathode (double-sided electrode loading of 35.2 mg/cm^2^), is shown in Figure 5a. An optical image of the assembled pouch cell is depicted in Figure S11. The pouch cell stayed at a capacity retention rate of 89.5% after 150 cycles with a discharge capacity of 0.17 Ah. This result underscores the robust electrochemical stability of the Li@P anode in a verification of the pouch cell. In Figure 5b, the capacity–voltage characteristics of the same pouch cell are displayed. The data reveal that the Li@P anode sustained a significant level of capacity retention and demonstrated consistent cycling performance through 140 cycles.

## 4. Conclusions

In this study, we introduced a facile red phosphorus-assisted solid-state friction method to in situ fabricate a uniform Li_3_P interphase on lithium metal surfaces. The Li_3_P interphase, with mixed ionic/electronic conductivity, can serve as an interface for Li^+^ deposition. The abundant lithiophilic Li_3_P phase in the modified layer can act as active sites for electrochemical reactions, inducing uniform lithium-ion deposition. Additionally, the Li_3_P phase exhibits high ionic conductivity, enabling the Li_3_P interphase to facilitate lithium-ion migration on the anode surface, which is conducive to the uniform distribution of lithium-ion flux at the anode interface. This, in turn, suppresses the growth of lithium dendrites and enhances the cycling stability of the lithium metal anode. Consequently, symmetric cells assembled with Li@P exhibited an ultra-long cycle life and lower polarization voltage. The merits of utilizing Li@P as anodes were convincingly showcased in high-load LFP||Li full cells and 0.2 Ah capacity Li||LCO pouch cells, where remarkable enhancements in battery cycle stability were achieved. This work provides valuable insights into the development and design of next-generation applicable LMBs.

## Data Availability

The original contributions presented in the study are included in the article/Supplementary Material, further inquiries can be directed to the corresponding authors.

## Supplementary Materials

The following supporting information can be downloaded at: https://www.mdpi.com/article/10.3390/ma18091930/s1, Figure S1. The X-ray diffraction (XRD) pattern of red phosphorus powder; Figure S2. SEM image of red phosphorus powder; Figure S3. A digital image of the prepared Li@P; Figure S4. The EDX mapping of Li@P; Figure S5. FTIR spectra of red phosphorus powder and Li@P; Figure S6. The contact angles of bare Li and Li@P with ester-based electrolyte; Figure S7. The charge–discharge profiles of LFP||Li at a 1 C rate after cycling; Figure S8. The charge–discharge profiles of LFP||Li at a 2 C rate after cycling; Figure S9. The charge–discharge profiles of LFP||Li at different rates after cycling; Figure S10. The charge-discharge profiles of LFP||Li at different rates after cycling. Figure S11. The digital image of the Li@P‖LiCoO_2_ pouch cell pouch; Table S1. A comparison of the impedance fitting results between the modified electrode (this study) and other reported surface-modified electrodes. References [12,23,36] are cited in the Supplementary Materials.

## References

[1] Chu S., Majumdar A. (2012). Opportunities and challenges for a sustainable energy future. Nature.

[2] Cheng X.-B., Zhang R., Zhao C.-Z., Zhang Q. (2017). Toward Safe Lithium Metal Anode in Rechargeable Batteries: A Review. Chem. Rev..

[3] Wang R., Cui W., Chu F., Wu F. (2020). Lithium metal anodes: Present and future. J. Energy Chem..

[4] Guo Y., Li H., Zhai T. (2017). Reviving Lithium-Metal Anodes for Next-Generation High-Energy Batteries. Adv. Mater..

[5] Zhao Q., Deng Y., Utomo N.W., Zheng J., Biswal P., Yin J., Archer L.A. (2021). On the crystallography and reversibility of lithium electrodeposits at ultrahigh capacity. Nat. Commun..

[6] Gao J., Chen C., Dong Q., Dai J., Yao Y., Li T., Rundlett A., Wang R., Wang C., Hu L. (2021). Stamping Flexible Li Alloy Anodes. Adv. Mater..

[7] Ou C.-H., Pan Y.-M., Tang H.-T. (2022). Electrochemically promoted N-heterocyclic carbene polymer-catalyzed cycloaddition of aldehyde with isocyanide acetate. Sci. China Chem..

[8] Zhao Z., Li B. (2021). Multi-storey corridor structured host for a large area capacity and high rate metallic lithium anode. Electrochim. Acta.

[9] Chazalviel J.N. (1990). Electrochemical aspects of the generation of ramified metallic electrodeposits. Phys. Rev. A.

[10] Xia S., Zhang X., Liang C., Yu Y., Liu W. (2020). Stabilized lithium metal anode by an efficient coating for high-performance Li–S batteries. Energy Storage Mater..

[11] Lu G., Nai J., Luan D., Tao X., Lou X.W. (2023). Surface engineering toward stable lithium metal anodes. Sci. Adv..

[12] Huang H., Liu S., Xie Y., Liu J., Shi C., Sun M., Peng H., Lan J., Deng Y.-P., Huang L. (2024). Constructing an Artificial Interface as a Bifunctional Promoter for the Li Anode and the NCM Cathode in Lithium Metal Batteries. J. Am. Chem. Soc..

[13] Wu F., Borodin O., Yushin G. (2017). In situ surface protection for enhancing stability and performance of conversion-type cathodes. MRS Energy Sustain..

[14] Ling C., Naren T., Liu X., Yang J., Xiao P., Wei W., Ji X., Kuang G.-C., Chen L. (2023). In-situ polymerization induced phase separation to develop high-performance self-healable polymeric electrolytes for lithium metal battery. Mater. Today Energy.

[15] Li M., Wang C., Davey K., Li J., Li G., Zhang S., Mao J., Guo Z. (2023). Recent progress in electrolyte design for advanced lithium metal batteries. SmartMat.

[16] Wang Y., Zheng C., Xie W., Liu X., Lu Y., Hou Y., Ma T., Yan Z., Chen J. (2024). Ether-Modified Nonflammable Phosphate Enabling Anion-Rich Electrolyte for High-Voltage Lithium Metal Batteries. Adv. Mater..

[17] Deng Y., Lu H., Cao Y., Xu B., Hong Q., Cai W., Yang W. (2019). Multi-walled carbon nanotube interlayers with controllable thicknesses for high-capacity and long-life lithium metal anodes. J. Power Sources.

[18] Pan L., Luo Z., Zhang Y., Chen W., Zhao Z., Li Y., Wan J., Yu D., He H., Wang D. (2019). Seed-Free Selective Deposition of Lithium Metal into Tough Graphene Framework for Stable Lithium Metal Anode. ACS Appl. Mater. Interfaces.

[19] Li S., Liu Q., Zhou J., Pan T., Gao L., Zhang W., Fan L., Lu Y. (2019). Hierarchical Co_3_O_4_ Nanofiber–Carbon Sheet Skeleton with Superior Na/Li-Philic Property Enabling Highly Stable Alkali Metal Batteries. Adv. Funct. Mater..

[20] Luo Z., Li S., Yang L., Tian Y., Xu L., Zou G., Hou H., Wei W., Chen L., Ji X. (2021). Interfacially Redistributed charge for robust lithium metal anode. Nano Energy.

[21] Sun Y., Zhao C., Adair K.R., Zhao Y., Goncharova L.V., Liang J., Wang C., Li J., Li R., Cai M. (2021). Regulated lithium plating and stripping by a nano-scale gradient inorganic–organic coating for stable lithium metal anodes. Energy Environ. Sci..

[22] Huang Y., Wang C., Lv H., Xie Y., Zhou S., Ye Y., Zhou E., Zhu T., Xie H., Jiang W. (2023). Bifunctional Interphase Promotes Li+ De-Solvation and Transportation Enabling Fast-Charging Graphite Anode at Low Temperature. Adv. Mater..

[23] Fu L., Wang X., Zhang B., Chen Z., Li Y., Sun Y. (2024). A Li3P nanoparticle dispersion strengthened ultrathin Li metal electrode for high energy density rechargeable batteries. Nano Res..

[24] Tu S., Zhang B., Zhang Y., Chen Z., Wang X., Zhan R., Ou Y., Wang W., Liu X., Duan X. (2023). Fast-charging capability of graphite-based lithium-ion batteries enabled by Li3P-based crystalline solid–electrolyte interphase. Nat. Energy.

[25] Li J., Liu D., Sun H., Qu D., Xie Z., Tang H., Liu J. (2023). Mixed ion-electron conducting Li3P for efficient cathode prelithiation of all-solid-state Li-ion batteries. SmartMat.

[26] Chen W., Salvatierra R.V., Li J.T., Luong D.X., Beckham J.L., Li V.D., La N., Xu J., Tour J.M. (2022). Brushed Metals for Rechargeable Metal Batteries. Adv. Mater..

[27] Wang H., Liu C., Wang H., Han X., Zhang S., Sun J., Zhang Y., Cao Y., Yao Y., Sun J. (2021). The synthesis of greenish phosphorus on carbon substrates. Chem. Commun..

[28] Chang W.C., Wu J.H., Chen K.T., Tuan H.Y. (2019). Red Phosphorus Potassium-Ion Battery Anodes. Adv. Sci..

[29] Zhao Q., Hao X., Su S., Ma J., Hu Y., Liu Y., Kang F., He Y.-B. (2019). Expanded-graphite embedded in lithium metal as dendrite-free anode of lithium metal batteries. J. Mater. Chem. A.

[30] Zhu P., Jiang Z., Sun W., Yang Y., Silvester D.S., Hou H., Banks C.E., Hu J., Ji X. (2023). Built-in anionic equilibrium for atom-economic recycling of spent lithium-ion batteries. Energy Environ. Sci..

[31] He X., Hao W., Shi Z., Tan Y., Yue X., Xie Y., Yan X., Liang Z. (2024). Colloid Electrolyte Containing Li3P Nanoparticles for Highly Stable 4.7 V Lithium Metal Batteries. ACS Nano.

[32] Zhang C., Lyu R., Lv W., Li H., Jiang W., Li J., Gu S., Zhou G., Huang Z., Zhang Y. (2019). A Lightweight 3D Cu Nanowire Network with Phosphidation Gradient as Current Collector for High-Density Nucleation and Stable Deposition of Lithium. Adv. Mater..

[33] Li L., Xu G., Zhang S., Dong S., Wang S., Cui Z., Du X., Wang C., Xie B., Du J. (2022). Highly Fluorinated Al-Centered Lithium Salt Boosting the Interfacial Compatibility of Li-Metal Batteries. ACS Energy Lett..

[34] Krauskopf T., Richter F.H., Zeier W.G., Janek J. (2020). Physicochemical Concepts of the Lithium Metal Anode in Solid-State Batteries. Chem. Rev..

[35] Long K., Huang S., Wang H., Wang A., Chen Y., Liu Z., Zhang Y., Wu Z., Wang W., Chen L. (2024). Green mechanochemical Li foil surface reconstruction toward long-life Li–metal pouch cells. Energy Environ. Sci..

[36] Lai Y., Zhang H., Xia G., Yu X. (2022). Long-term stable Li metal anode enabled by strengthened and protected lithiophilic LiZn alloys. J. Power Sources.

[37] Long K., Liu X., Yang J., Wang H., Wang A., Chen Y., Mei L., Zhang Y., Wu Z., Wang W. (2024). Homogeneously Planar-Exposure LiB Fiber Skeleton Toward Long-Lifespan Practical Li Metal Pouch Cells. Small.

[38] Xiong X., Yan W., Zhu Y., Liu L., Fu L., Chen Y., Yu N., Wu Y., Wang B., Xiao R. (2022). Li_4_Ti_5_O_12_ Coating on Copper Foil as Ion Redistributor Layer for Stable Lithium Metal Anode. Adv. Energy Mater..

[39] Zhang T., Lu H., Yang J., Xu Z., Wang J., Hirano S.-i., Guo Y., Liang C. (2020). Stable Lithium Metal Anode Enabled by a Lithiophilic and Electron/Ion Conductive Framework. ACS Nano.

[40] Lin L., Liu F., Zhang Y., Ke C., Zheng H., Ye F., Yan X., Lin J., Sa B., Wang L. (2022). Adjustable Mixed Conductive Interphase for Dendrite-Free Lithium Metal Batteries. ACS Nano.

[41] Long K., Huang S., Wang H., Jin Z., Wang A., Wang Z., Qing P., Liu Z., Chen L., Mei L. (2023). High interfacial capacitance enabled stable lithium metal anode for practical lithium metal pouch cells. Energy Storage Mater..

