# Supplementary material for: A Lithiophilic Artificial Li3P Interphase with High Li-Ion Conductivity via Solid-State Friction for Lithium Metal Anodes
Source: Materials (Basel). 2025 Apr 24;18(9):1930. doi: 10.3390/ma18091930 (PMC12073039; doi:10.3390/ma18091930)
Supplement: Supplementary file 1 [file materials-18-01930-s001.zip › materials-3550803-supplementary.pdf]

Supporting Information

A Lithiophilic Artificial Li_3P Interphase with High Li-Ion Conductivity via Solid-State Friction for Lithium Metal Anodes

Haoling Liu[1], Wen Pan[1], Bo Xiao[1], Yunke Jin[1], Kun Li[1], An Wang[1], Huimiao Li[1], Zhibin Wu[1], Yuejiao Chen[1], Shaozhen Huang [1]*, Lin Mei[1]*, Libao Chen[1]*

[1] *State Key Laboratory of Powder Metallurgy, Central South University, Changsha, 410083, P. R. China.*

**Corresponding Author: Shaozhen Huang (hsz13959391336@csu.edu.cn);* Lin Mei *(meilin@csu.edu.cn);* Libao Chen (lbchen@csu.edu.cn)

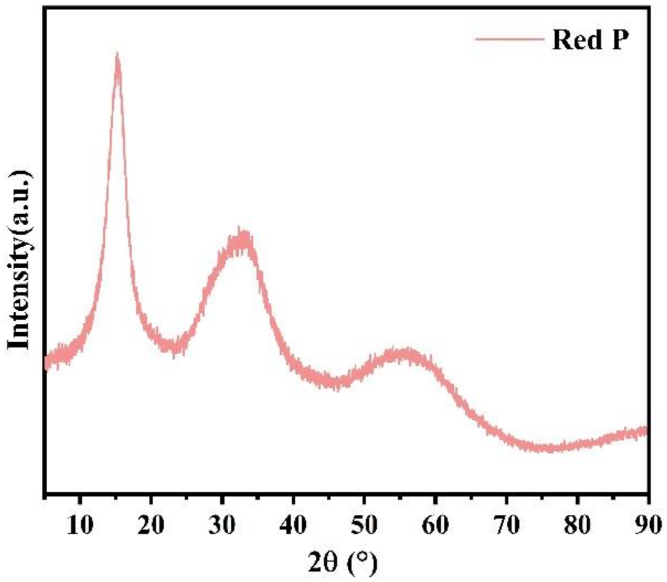

Figure S1. The X-ray diffraction (XRD) pattern of red phosphorus powder.

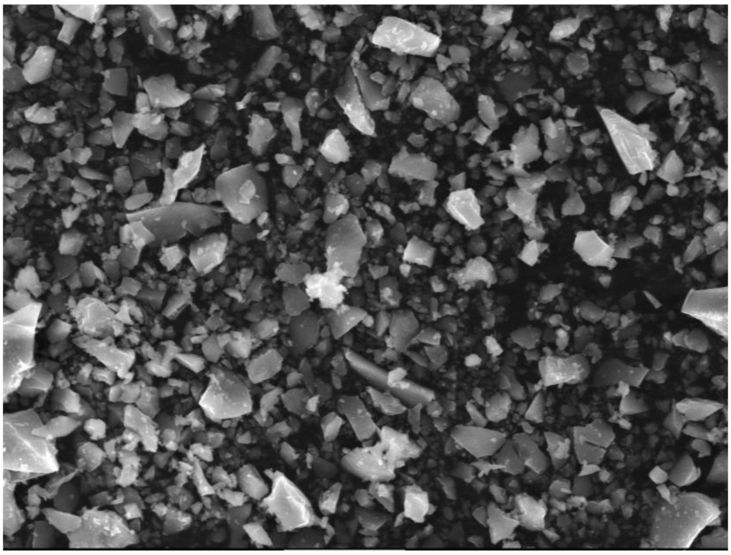

Figure S2. SEM image of red phosphorus powder.

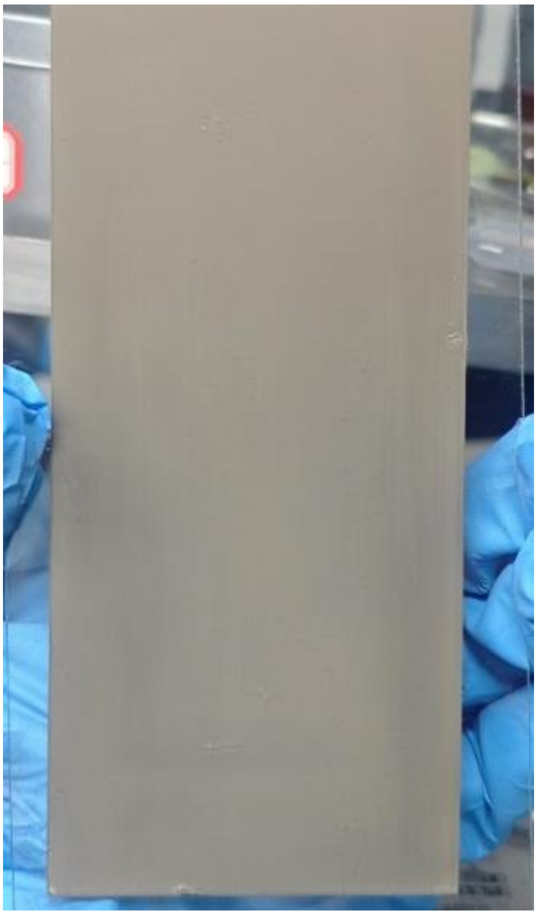

Figure S3. The digital image of the prepared Li@P.

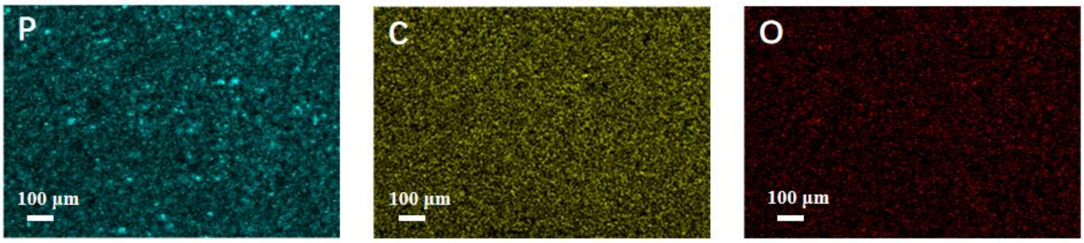

Figure S4. The EDX mapping of Li@P

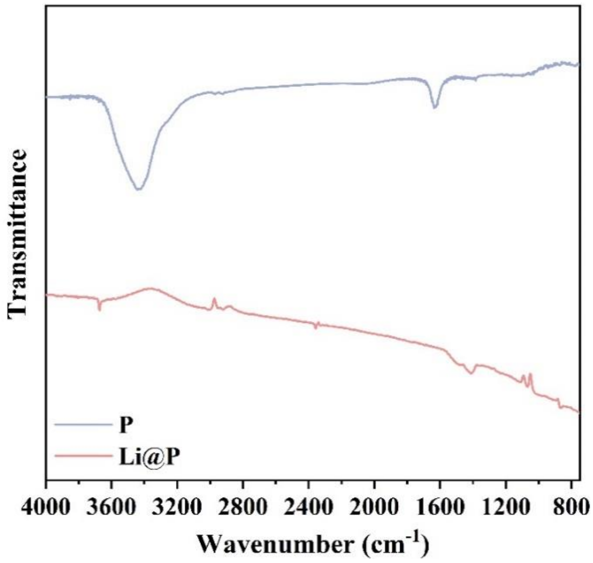

Figure S5. FTIR spectra of red phosphorus powder and Li@P.

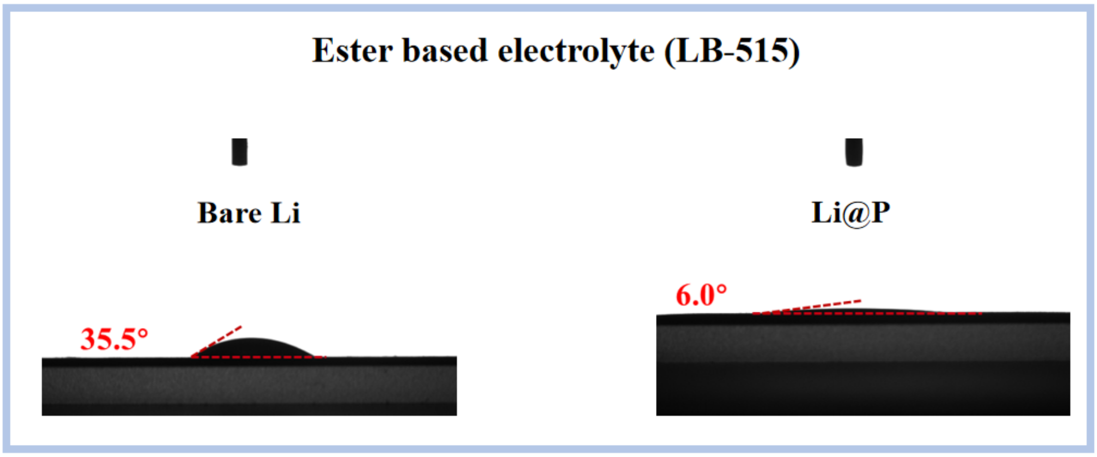

Figure S6. The contact angles of Bare Li and Li@P with ester based electrolyte.

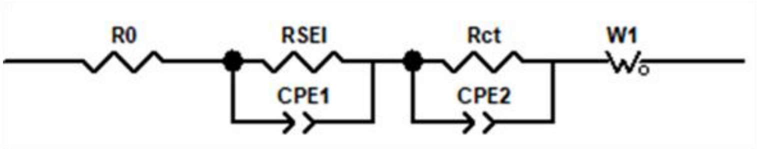

Figure S7. The equivalent circuit of Nyquist plot.

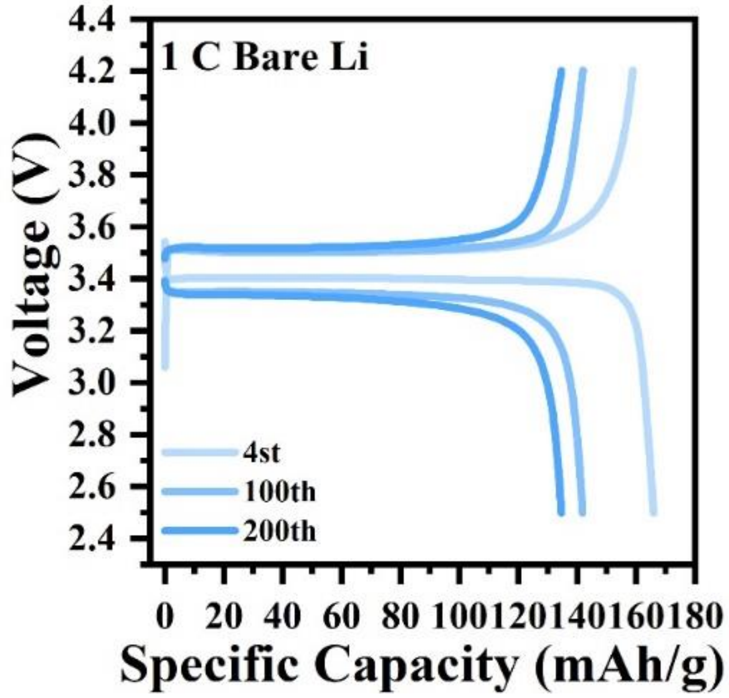

Figure S8. The charge-discharge profiles of LFP||Li at 1 C rate after cycling

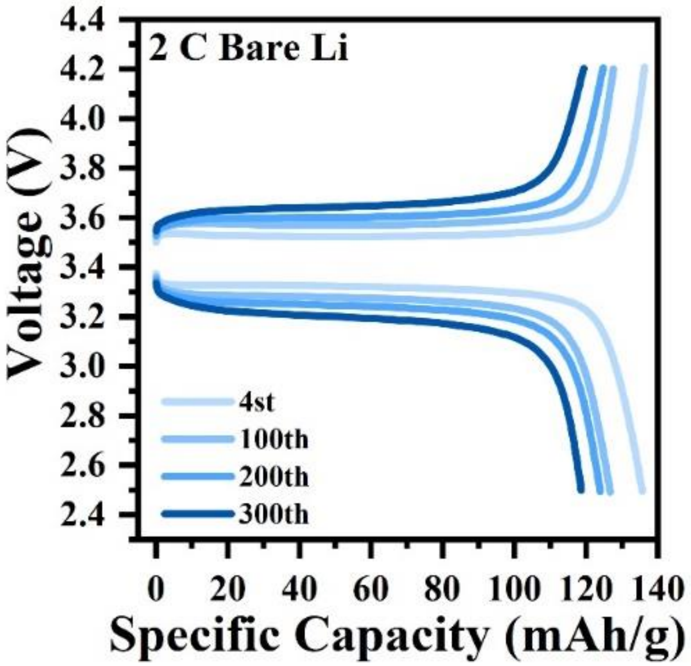

Figure S9. The charge-discharge profiles of LFP||Li at 2 C rate after cycling

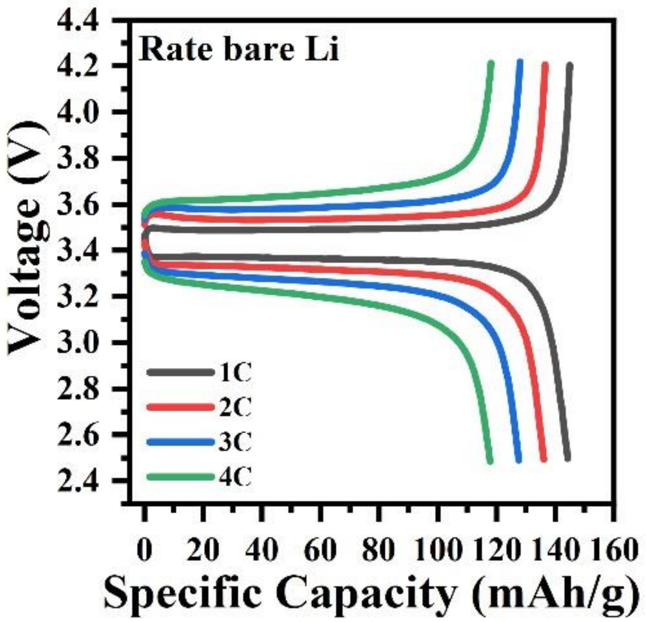

Figure S10. The charge-discharge profiles of LFP||Li at different rates after cycling.

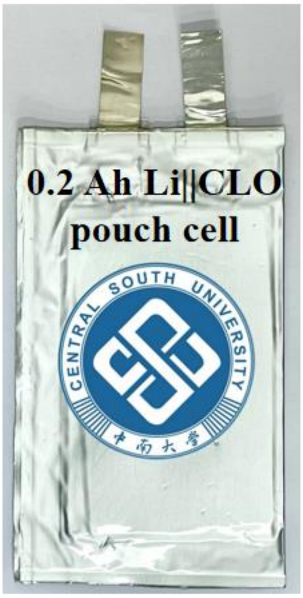

Figure S11. The digital image of Li@P||$LiCoO_2$ pouch cell pouch.

Table S1 A comparison of the impedance fitting results between the modified electrode (this study) and other reported surface-modified electrodes.

	**R_{SEI} (ohm)**	**R_{ct} (ohm)**	**Ref**
Li@P	8.3	24.7	（this work）
Bare Li	209.7	223.7	（this work）
Work 1	2.7	205.4	[1]
Work 2		41	[2]
Work 3		42.75	[3]

[1] H. Huang, S. Liu, Y. Xie, J. Liu, C. Shi, M. Sun, H. Peng, J. Lan, Y.-P. Deng, L. Huang, S.-G. Sun, Constructing an Artificial Interface as a Bifunctional Promoter for the Li Anode and the NCM Cathode in Lithium Metal Batteries, Journal of the American Chemical Society 146 (2024) 31137-31149.
[2] Y. Lai, H. Zhang, G. Xia, X. Yu, Long-term stable Li metal anode enabled by strengthened and protected lithiophilic LiZn alloys, Journal of Power Sources 543 (2022).
[3] L. Fu, X. Wang, B. Zhang, Z. Chen, Y. Li, Y. Sun, A Li3P nanoparticle dispersion strengthened ultrathin Li metal electrode for high energy density rechargeable batteries, Nano Research 17 (2024) 4031-4038.
